# Early detection of pulmonary mucormycosis using microbial cell-free DNA sequencing of bronchoalveolar lavage fluid in neutropenic Hosts: A report of two cases

**DOI:** 10.1016/j.mmcr.2026.100795

**Published:** 2026-05-08

**Authors:** Yupeng Liu, Julia S. Zinn, Kevin M. Grudzinski

**Affiliations:** a Department of Medicine, Northwestern University Feinberg School of Medicine, Chicago, IL, United States; b Medical Science Liaison, Karius Inc, Redwood City, CA, United States; c Division of Pulmonary and Critical Care, Department of Medicine, Northwestern University Feinberg School of Medicine, Chicago, IL, United States

**Keywords:** Mucormycosis, Fungal, Microbiologic assay, Pulmonology

## Abstract

Mucormycosis is an aggressive fungal infection characterized by angioinvasion, tissue necrosis, and high mortality rates. Microbial cell-free DNA (mcfDNA) sequencing provides rapid, non-invasive detection of microbial genetic material to guide diagnosis and treatment. We describe 2 cases of immunocompromised patients with invasive mucormycosis wherein mcfDNA sequencing significantly expedited diagnosis, allowing early surgical intervention and antimicrobial treatment.

## 1 Introduction

Mucormycosis is a rapidly progressive fungal infection characterized by high mortality rates [1]. Its pathophysiology is driven by angioinvasion and vascular thrombosis leading to extensive tissue necrosis and impaired drug delivery. Early diagnosis and aggressive surgical debridement of necrotic tissue is quintessential for survival [2,3]. While traditional diagnostic methods like microscopy, culture, and histopathology are standard of care, *Mucorales* are notoriously difficult to isolate. Culture confirmation, which is essential for targeted treatment, is limited by organism fragility during specimen preparation and long incubation periods [3,4]. Sensitivity of cultures from blood, sputum, and bronchoalveolar lavage fluid (BALF) remain exceedingly low, approximating 50% [4].

Recent years have seen increased utilization of molecular diagnostic methods, including polymerase chain reaction (PCR) sequencing. However, microbiologic PCR testing has its own limitations, including false-positive detections, targeted design for specific targets, and challenges with polymicrobial detection. Metagenomic next-generation sequencing of microbial cell-free DNA (mcfDNA), a high-throughput molecular diagnostic tool that detects and quantifies circulating DNA fragments from over 1000 bacteria, fungi, viruses, and parasites simultaneously, offers rapid detection with high sensitivity from non-invasive sampling techniques [5]. In clinical practice, mcfDNA sequencing serves as a pathogen-agnostic test with a broad differential diagnosis for infections, especially for patients with contraindications to invasive sampling. To date, there is no consensus guideline on how to integrate mcfDNA sequencing into standard diagnostic evaluation for suspected mucormycosis in neutropenic patients. Furthermore, the limited extant literature evaluating mcfDNA sequencing for invasive fungal disease in neutropenic patients primarily included *Aspergillus* and other molds without *Mucorales*-specific performance [6].

We present two cases of pulmonary mucormycosis, one with extrapulmonary infection, diagnosed by BALF-mcfDNA sequencing in immunocompromised patients with neutropenia. In both cases, mcfDNA sequencing yielded pathogen identification within 48 hours, whereas pathogen confirmation via *Mucorales* PCR and tissue cultures required between 7 and 31 days to result. Subsequent early surgical intervention provided histopathological confirmation of extensive fungal invasion. The objective of this case report is to demonstrate the timeline of species-level identification of mucormycosis by mcfDNA sequencing in neutropenic patients at risk for invasive fungal infection, contextualizing the assay findings in the setting of their clinical management and disease course.

## 2 Case presentation

### 2.1 Case 1

**Fig. 1:**
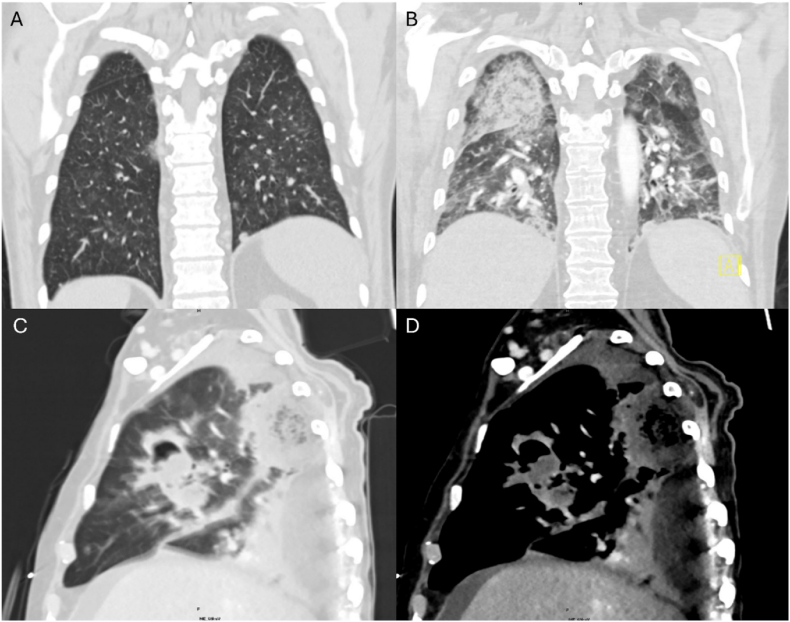
CT imaging of progressive pulmonary mucormycosis for Case 1. **1A** is a coronal view of the patient's bilateral lung fields from his first hospitalization, notable for scattered groundglass nodular opacities. **1B** depicts bilateral lung fields from a CT angiogram one month later during his second hospitalization. **1C** and **1D** were obtained 3 weeks later and depict the same sagittal plane of the right lung showing severe architectural distortion with a large masslike consolidation in the right lung apex and a large right middle lobe cavitary mass in lung and soft tissue windows, respectively.

A 58-year-old male with a history of hemophagocytic lymphohistiocytosis (HLH) on ruxolitinib, emapalumab, and anakinra presented to the hospital with neutropenic fever and cough. Computed tomography (CT) imaging of the chest showed scattered subcentimeter ground-glass opacities (Fig. 1A). Bronchoscopy with bronchoalveolar lavage (BAL) was performed with negative bacterial and fungal cultures, Aspergillus galactomannan, and multiplex molecular PCR panel for pneumonia. He received one week of empiric antibiotics with cefepime and azithromycin. Given high clinical suspicion for HLH flare rather than acute infection, he was started on etoposide and dexamethasone with clinical improvement, and one week after admission he was discharged home.

The following month, the patient presented again for 1 week of dyspnea. Upon admission, the patient was afebrile and hemodynamically stable. Complete blood count (CBC) with differential was notable for pancytopenia with WBC 0.5*10^3^/μL, absolute neutrophil count 100/μL, hemoglobin 8.1g/dL, platelets 33*10^3^/μL. Preliminary infectious workup with blood cultures and antigen testing for *Streptococcus pneumonia*, *Legionella*, *Histoplasma*, and *Blastomycoses* were all negative. However, CT chest revealed progressive multifocal opacities (Fig. 1B), so he was empirically started on cefepime and azithromycin.

He underwent urgent bronchoscopy with BAL three days after admission. Conventional infectious studies and mcfDNA sequencing were sent from BALF. McfDNA sequencing returned 48 hours later with identification of multiple opportunistic pathogens including *Rhizopus arrhizus*, *Cunninghamella*, and *Aspergillus fumigatus*. The patient was started on intravenous amphotericin B at 5 mg/kg every 24 hours and oral posaconazole 300 mg twice daily for pulmonary mucormycosis. After consideration of *Cunninghamella* detection, he was also started on oral terbinafine hydrochloride at 250 mg daily. *Stenotrophomonas maltophilia* and *Prevotella melaninogenica* were also detected but were considered commensals and not treated due to their lower pathogenic potential and extant literature supporting their presence as normal respiratory tract flora [7]. *Mucorales* PCR from BALF was sent out for processing and returned positive 11 days later. BALF aerobic and anaerobic cultures grew *Stenotrophomonas maltophilia* (3000 CFU/mL), while acid-fast bacilli (AFB) culture and multiplex PCR panel for pneumonia were negative. BALF fungal culture remained pending.

Upon evaluation for pulmonary resection, it was determined that surgical risk was prohibitive due to overwhelming multifocal disease. Evaluation for extrapulmonary mucormycosis infection with CT sinuses revealed severe diffuse sinusitis. Bedside nasal endoscopy showed bilateral eschar and mucosal pallor. The patient underwent urgent endoscopic ethmoidectomy and debridement of the sinuses, turbinates, and septum with resection of necrotic tissue, which yielded histopathological confirmation of fungal hyphae with Grocott's Methenamine Silver (GMS) stain showing invasion of blood vessels, soft tissue, and bone. Nasal tissue cultures were obtained and grew *Fusarium* species after 7 days.

Two days after his initial surgical debridement, repeat CT sinuses showed progressive severe opacification of the bilateral paranasal sinuses, and the patient underwent repeat surgical debridement. This time, GMS staining did not show definitive histopathologic evidence of fungal elements. However, post-operative CT brain and angiography demonstrated findings consistent with fungal angioinvasion, and MRI brain revealed multiple small acute infarcts likely secondary to infectious emboli.

Two weeks later, CT chest revealed increased bilateral mass-like consolidations with new cavitation (Fig. 1C/1D), and CT sinuses showed near-complete opacification of the bilateral sinuses. Upon discussion between the patient and multiple teams regarding his poor prognosis and the lack of further treatment options, the patient elected to pursue hospice care and passed away soon after. Thirty-one days after his initial bronchoscopy, BALF fungal culture grew *Cunninghamella*.

### 2.2 Case 2

A 36-year-old male with a history of acute myeloid leukemia (AML) presented to the hospital for a planned chemotherapy regimen of FLAG-IDA and venetoclax. Admission CBC with differential was notable for pancytopenia with WBC 0.7*10^3^/μL, absolute neutrophil count 100/μL, hemoglobin 7.1g/dL, platelets 21*10^3^/μL. Two weeks after admission, he developed neutropenic fever to 101.3 °F and started on piperacillin-tazobactam. At this time, he had no localizing signs or symptoms of infection. The fever persisted, and broad infectious workup returned with negative blood cultures and antigen testing for *Streptococcus pneumonia*, *Legionella*, *Histoplasma*, and *Blastomycoses*.

**Fig. 2:**
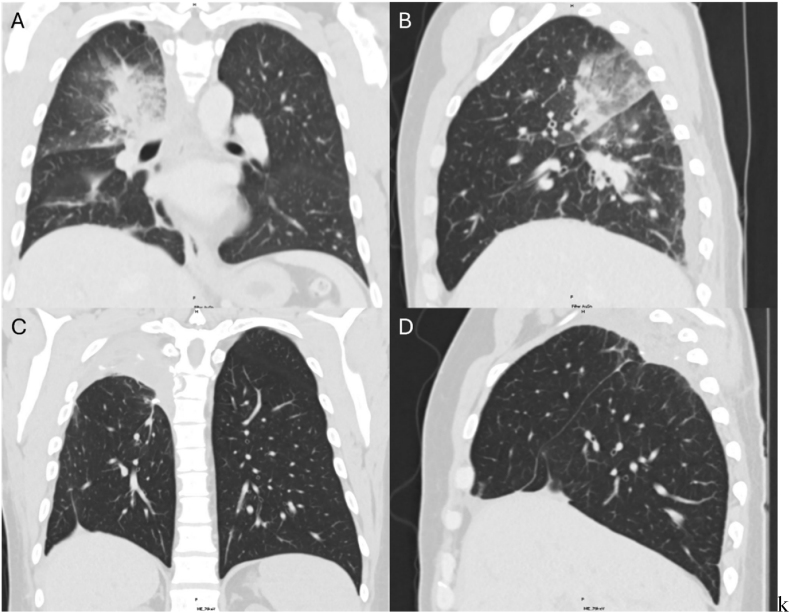
Right pulmonary mucormycosis before and after surgical lung resection. **2A** is a coronal view of the patient's bilateral lung fields from his initial infectious workup, notable for consolidation in the perihilar posterior right upper lobe and superior segment of the right lower lobe with bronchial wall thickening and surrounding groundglass opacities. **2B** depicts the right lung pathology from the same CT in a sagittal view. **2C** and **2D** are views from CT chest obtained approximately 2 months following right upper lobectomy and lower lobe segmentectomy showing a coronal view of the bilateral lung fields and a sagittal view of the right lung, respectively.

CT chest, abdomen, and pelvis revealed a new right upper lobe peribronchial consolidation (Fig. 2). Due to concern for atypical or fungal infection, bronchoscopy with BAL was performed with conventional infectious studies and mcfDNA sequencing sent from BALF. Within 48 hours, mcfDNA sequencing returned with identification of *Rhizomucor pusillus*, and IV amphotericin was initiated. Also detected were *Campylobacter gracilis*, *Prevotella melaninogenica*, and *Schaalia odontolytica*, which were regarded as were also detected but were considered commensals and not treated due to their lower pathogenic potential and prevalence as respiratory flora [7]. *Mucorales* PCR was sent out for processing and returned positive 7 days later. BALF aerobic and anaerobic cultures grew *Schaalia* species (10,000 CFU/mL), while fungal culture, AFB culture, Aspergillus galactomannan antigen, and multiplex molecular PCR panel for pneumonia were negative.

**Fig. 3:**
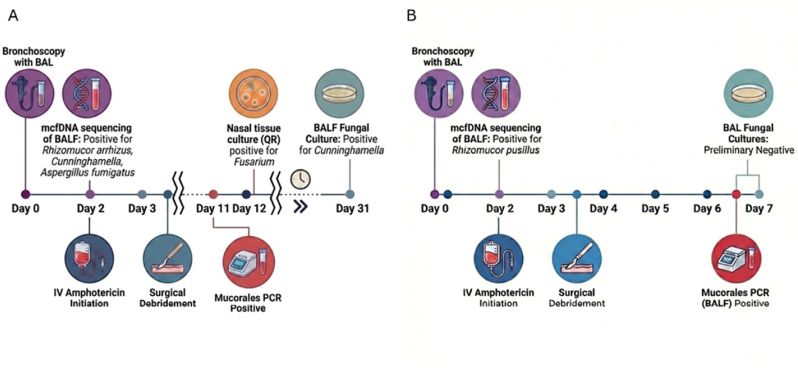
Timeline of diagnostic testing and clinical interventions for Case 1 (Fig. 3A) and Case 2 (Fig. 3B).

The next day, the patient developed pleuritic chest pain and small-volume hemoptysis. Repeat CT imaging revealed increased consolidations of the right upper lobe compatible with pulmonary mucormycosis (Fig. 2). The patient underwent surgical resection with an open right upper lobectomy and right lower lobe segmentectomy with an intercostal muscle flap. Intraoperative findings were notable for significant adhesions, complete upper lobe consolidation, and extensive necrosis. Histopathological analysis of the resected lung tissue revealed lung parenchyma with hemorrhage, granulation tissue, and foci of necrosis with clusters of fungal hyphae suggestive of mucormycosis. Tissue from the chest wall debridement demonstrated foci of necrosis without fungal elements.

The patient received a 2-week course of intravenous amphotericin B at 7.5 mg/kg every 24 hours and was transitioned to oral posaconazole 300 mg daily afterwards which was continued indefinitely given the patient's concurrent neutropenia. Serial chest radiographs following the patient's surgical resection were stable. Intraoperative nasal tissue cultures remained without growth. He completed his chemotherapy regimen with bone marrow biopsy confirming no residual disease. Six weeks after admission, the patient was discharged home.

## 3 Discussion

McfDNA sequencing offers significant potential to expedite diagnosis and treatment initiation for mucormycosis. In both cases, BALF-mcfDNA sequencing provided rapid, actionable microbiological data within 48 hours without requiring lung biopsies, which would have involved significant risk due to severe thrombocytopenia. This is consistent with literature suggesting that mcfDNA sequencing can identify pathogenic mold up to 3 weeks before clinical diagnosis of pulmonary mold infections [8]. This intrinsic advantage of mcfDNA sequencing may also improve its ability to detect multiple concomitant mucormycoses or coinfection with other fungal organisms such as *Aspergillus* spp. Which can be seen in up to a third of confirmed mucormycosis cases, including our first case [9].

In **Case 1**, mcfDNA sequencing detection was the turning point that prompted sinus CT and endoscopy despite the absence of sinusitis, which yield histopathologic confirmation of mucormycosis. In comparison, *Mucorales* PCR returned positive after 11 days, although under ideal conditions with on-site analyzers the turnaround time may be closer to 2-5 days, but ultimately would not yield species level detection [10]. BALF fungal culture speciated *Cunninghamella* after 29 days but failed to detect *Rhizopus arrhizus*. The timeline of events highlights the limitations in speed and sensitivity of current diagnostic techniques. Ultimately, mcfDNA sequencing detection of *Mucorales* enabled timely antifungal initiation and surgical intervention prior to any clinical manifestations of extrapulmonary rhinocerebral disease.

While surgical nasal tissue cultures grew *Fusarium* species, this was not detected on BALF mcf-DNA sequencing. This most likely reflects localized soft tissue infection by *Fusarium* without pulmonary involvement or angioinvasion, for which plasma mcfDNA sequencing could potentially be more sensitive. In the absence of BALF mcfDNA sequencing, the positive *Fusarium* tissue culture would have been the only fungal microbial data available as *Mucorales* PCR did not result until later. Consequentially, reliance upon tissue culture data alone may have steered antifungal selection towards *Fusarium* coverage with voriconazole, which would have failed to provide antimicrobial activity against mucormycosis.

**Case 2** describes pulmonary mucormycosis diagnosed by mcfDNA sequencing with BALF in a patient with neutropenic fever of unknown origin who lacked pulmonary symptoms such as cough, dyspnea, or increased sputum production. By the time he developed pleurisy and hemoptysis as the infection progressed, mcfDNA sequencing had detected *Rhizomucor pusillus* and surgical resection was already planned. Unlike **Case 1** where BALF fungal culture eventually speciated *Cunninghamella*, no cultures from **Case 2** detected *Mucorales*. The high sensitivity and fast turnaround of mcfDNA sequencing enabled early diagnosis and source control prior to disease progression.

A major limitation of mcfDNA sequencing is the potential to detect genetic material from non-infectious colonizers or contaminants [11]. Both of our cases detected non-infectious microbial genetic content, highlighting the complexity behind deciding upon treatment initiation versus deferral. Careful clinical correlation with underlying risk factors, relevant exposures, imaging, and physical exam is necessary to distinguish mucormycosis colonization from infection. In both cases, the index of suspicion for active infection was high due to neutropenia, respiratory symptoms, and compatible imaging findings. Subsequent operative interventions were targeted to the affected regions on imaging, ultimately yielding histopathologic confirmation of fungal invasion.

Another limitation of mcfDNA sequencing is the higher cost relative to multiplex PCR panels, and particularly in comparison to single-target PCR assays. The cost expense involved in mcfDNA sequencing may present a barrier restricting accessibility. Cost-benefit analyses are required to better characterize the financial and resource expenses of this platform on health systems. Additionally, the small sample size reported in this case report of two patients limits the ability to conclude that mcfDNA sequencing should be integrated into routine diagnostic workflows.

In summary, our cases illustrate the ability of mcfDNA sequencing to expedite mucormycosis diagnosis and treatment. Of note, we described a case of pulmonary mucormycosis in a young patient with AML where mcfDNA sequencing detected mucormycosis within 48 hours, enabling surgical planning prior to emergence of pulmonary symptoms. Following lung resection, the patient completed his chemotherapy course and discharged home without complication. The diagnostic potential of mcfDNA sequencing may offer a new path forward in managing patients with mucormycosis by allowing detection and treatment initiation prior to disease progression and manifestation of clinical symptoms. However, further studies are needed to analyze the sensitivity and specificity of mcfDNA sequencing assays in detecting mixed mold infections, the performance of these assays on blood versus BALF, comparisons with conventional single and multiplex PCR, and the optimal timing of testing relative to symptomatology and antimicrobial initiation.

## CRediT authorship contribution statement

**Yupeng Liu:** Writing – review & editing, Writing – original draft, Visualization, Investigation, Formal analysis, Data curation. **Julia S. Zinn:** Writing – review & editing, Visualization, Validation, Software, Data curation, Conceptualization. **Kevin M. Grudzinski:** Writing – review & editing, Supervision, Investigation, Data curation, Conceptualization.

## Ethical form

Ethical form submitted separately. Consent was obtained from the patient and/or next of kin for authorization of use of their de-identified medical information for research purposes.

## Declaration of generative AI and AI-assisted technologies in the manuscript preparation process

Statement: During the preparation of this work the author(s) Google Gemini to generate Fig. 3, a timeline depicting the sequence of events for the two clinical cases. After using this tool/service, the author(s) reviewed and edited the content as needed and take(s) full responsibility for the content of the published article.

## Conflict of interest

No funding sources were obtained for this project. Julia Zinn is a medical science liaison for Karius Inc.

## References

[1] Global guideline for the diagnosis and management of mucormycosis: an initiative of the European confederation of medical Mycology in cooperation with the Mycoses Study Group Education and Research Consortium - PubMed [Internet]. [cited 2026 Feb 11]. https://pubmed.ncbi.nlm.nih.gov/31699664/.

[2] Skiada A., Pavleas I., Drogari-Apiranthitou M. (2020 Nov 2). Epidemiology and diagnosis of mucormycosis: an update. J. Fungi.

[3] Walsh T.J., Gamaletsou M.N., McGinnis M.R., Hayden R.T., Kontoyiannis D.P. (2012 Feb). Early clinical and laboratory diagnosis of invasive pulmonary, extrapulmonary, and disseminated mucormycosis (zygomycosis). Clin Infect Dis Off Publ Infect Dis Soc Am..

[4] Lackner M., Caramalho R., Lass-Flörl C. (2014). Laboratory diagnosis of mucormycosis: current status and future perspectives. Future Microbiol..

[5] Colombo A.L., de Almeida Júnior J.N., Slavin M.A., Chen S.C.A., Sorrell T.C. (2017 Nov). Candida and invasive mould diseases in non-neutropenic critically ill patients and patients with haematological cancer. Lancet Infect. Dis..

[6] Huygens S., Schauwvlieghe A., Wlazlo N., Moors I., Boelens J., Reynders M. (2024 May 3). Diagnostic value of microbial cell-free DNA sequencing for suspected invasive fungal infections: a retrospective multicenter cohort Study. Open Forum Infect. Dis..

[7] Manual of clinical microbiology - national Library of Medicine Institution. https://catalog.nlm.nih.gov/discovery/fulldisplay/alma9917459033406676/01NLM_INST:01NLM_INST.

[8] Heldman M.R., Ahmed A.A., Liu W., Vo A., Keane-Candib J., Stevens-Ayers T. (2024 Feb 14). Serial quantitation of plasma microbial cell-free DNA before and after diagnosis of pulmonary invasive mold infections after hematopoietic cell transplant. J. Infect. Dis..

[9] Ra S.H., Kim J.Y., Song J.S., Jang H.M., Chang E., Bae S. (2024 Aug 2). Aspergillosis coinfection in patients with proven mucormycosis. Med. Mycol..

[10] Mucorales by PCR ARUP laboratories Test directory. https://ltd.aruplab.com/Tests/Pub/3000352.

[11] Kowarsky M., Camunas-Soler J., Kertesz M., De Vlaminck I., Koh W., Pan W. (2017 Sep 5). Numerous uncharacterized and highly divergent microbes which colonize humans are revealed by circulating cell-free DNA. Proc. Natl. Acad. Sci. U. S. A.

